# Achilles Tendon Xanthoma Thickness and Carotid Intima-Media Thickness in a Patient With Heterozygous Familial Hypercholesterolemia on PCSK9 Inhibition: A Case Report and Literature Review

**DOI:** 10.7759/cureus.10497

**Published:** 2020-09-16

**Authors:** Loba Alam, Glenmore Lasam, Robert Fishberg

**Affiliations:** 1 Internal Medicine, Overlook Medical Center, Summit, USA; 2 Cardiology, Atlantic Health System Overlook Medical Center, Summit, USA

**Keywords:** pcsk9 inhibitor, familial hypercholesterolemia, carotid intima-media thickness, ultrasound guided imaging, achilles tendon xanthoma

## Abstract

Ultrasound-guided measurement of carotid intima-media thickness can be used as a surrogate marker to predict future risk of atherosclerotic cardiovascular disease, and to understand the efficacy of lipid-lowering drugs. Aggressive lipid-lowering drugs such as proprotein convertase subtilisin/kexin type 9 (PCSK9) inhibitors have been shown to reduce carotid artery plaque burden, total cholesterol, and low-density lipoprotein-c in patients with heterozygous familial hypercholesterolemia (FH). We describe a patient with heterozygous FH treated with PCSK9 inhibitor over the course of two years, and the drug's impact on carotid intima-media thickness, Achilles tendon thickness, and cardiovascular disease risk reduction.

## Introduction

Ultrasound-guided imaging is one of the most widely used atherosclerotic imaging techniques. Ultrasound-guided measurement of Achilles tendon thickness (ATT) has been shown to correlate with disease severity and is considered as an important diagnostic criterion for familial hypercholesterolemia (FH) [1,2]. However, regression of ATT with lipid-lowering drugs has not consistently proven to be a good surrogate marker for disease progression or drug efficacy. However, ultrasound-guided measurement of carotid intima-media thickness (CIMT) and subsequent plaque regression with lipid-lowering drugs has shown to be among the most useful surrogate markers of atherosclerotic cardiovascular disease (ASCVD) risk reduction and drug efficacy [3,4]. The aim of this case report is to evaluate clinical and ultrasonographic changes of CIMT and ATT in a patient with virulent subtype of FH who was treated with proprotein convertase subtilisin-kexin 9 (PCSK9) inhibitor. At a two-year follow-up, we aim to correlate ultrasonographic changes of CIMT with the future risk of ASCVD and the efficacy of PCSK9 inhibition.

## Case presentation

A 27-year-old male with a past medical history significant for FH diagnosed in 2006 with a particularly virulent genetic form of heterozygous FH involving mutations in low-density lipoprotein (LDL) receptor with Gln125 variant and apolipoprotein E (apoE) haplotype E3/E4 and severe triple vessel coronary artery disease status post coronary artery bypass graft surgery in 2017 was initiated on aggressive lipid-lowering regimen to prevent further ASCVD. He was previously found to be intolerant to several statins and was started on the maximum tolerated dose of simvastatin, ezetimibe, and alirocumab, a PCSK9 inhibitor. 

**Figure 1:**
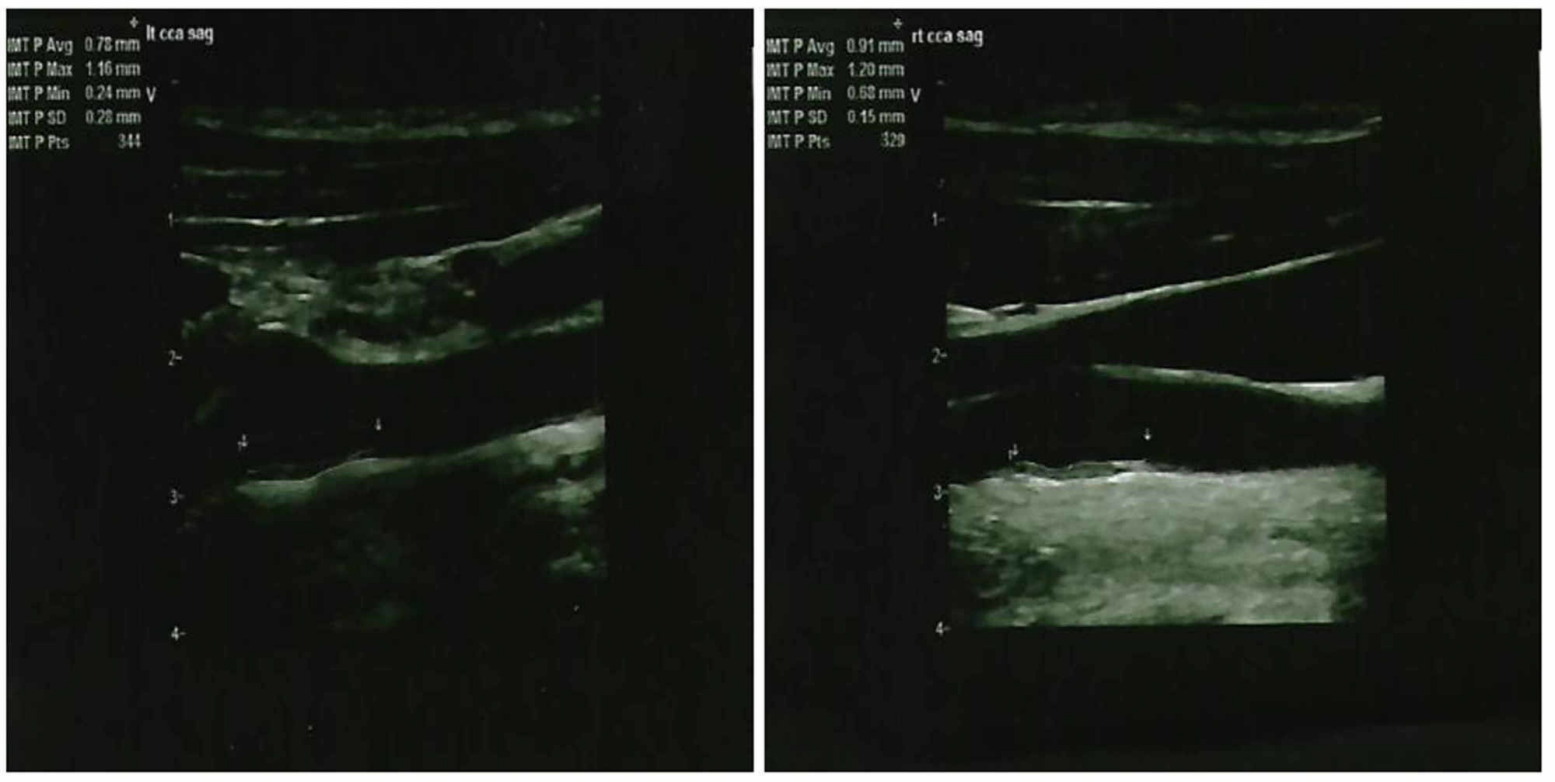
Ultrasound showing the left common carotid intima-media thickness (left panel) and right common carotid intima-media thickness (right panel) in 2017.

**Figure 2:**
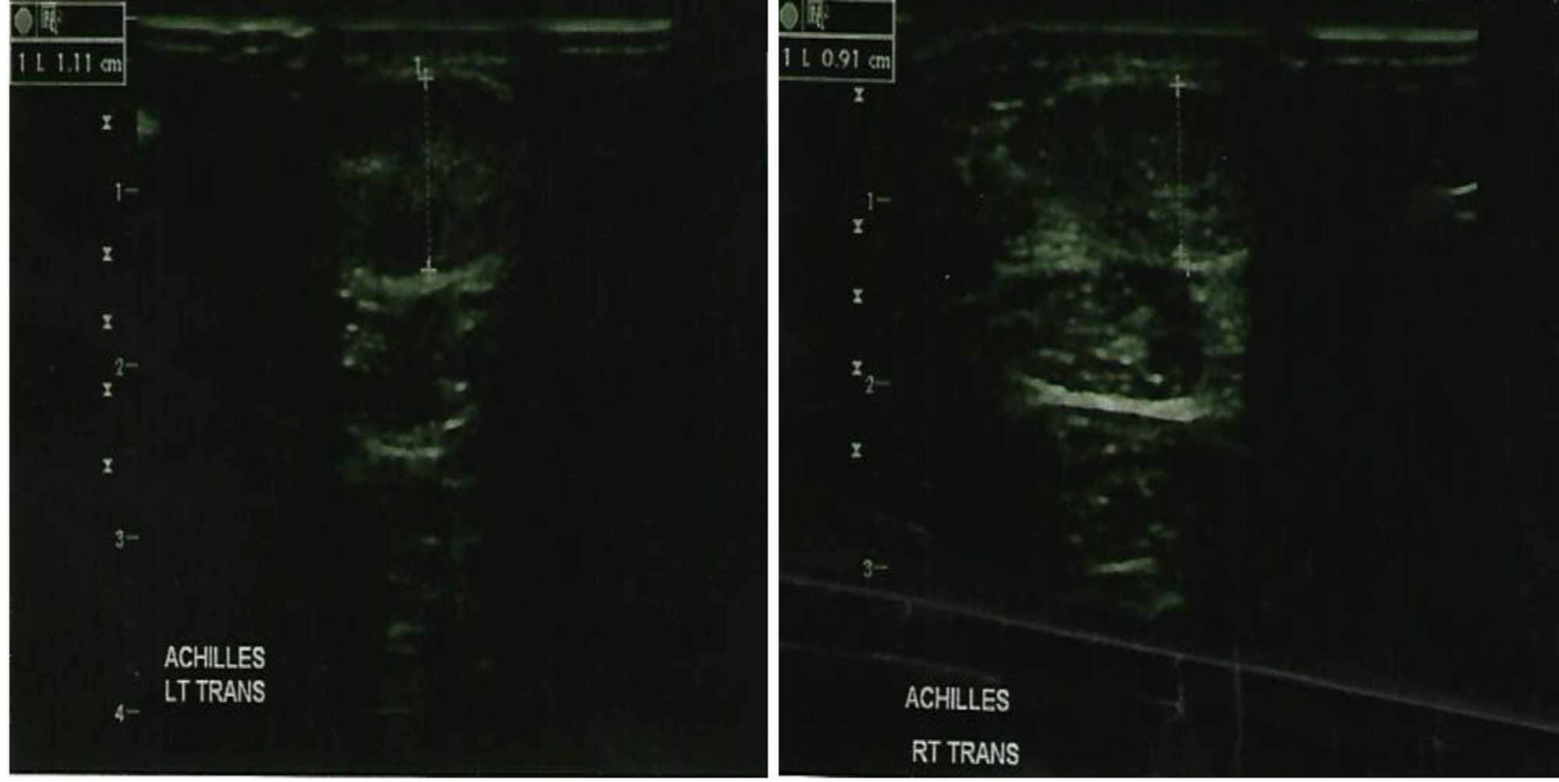
Ultrasound showing the left Achilles tendon thickness (left panel) and right Achilles tendon thickness (right panel) in 2017.

At that time in 2017, ultrasound of bilateral common carotids showed CIMT to be 0.91mm on right and 0.78mm on left (Figure 1), and ultrasound of bilateral Achilles tendon showed ATT to be 0.91cm on right and 1.11cm on left (Figure 2) [5]. He had a total cholesterol of 419mg/dl and LDL-c of 346mg/dl [5]. 

**Figure 3:**
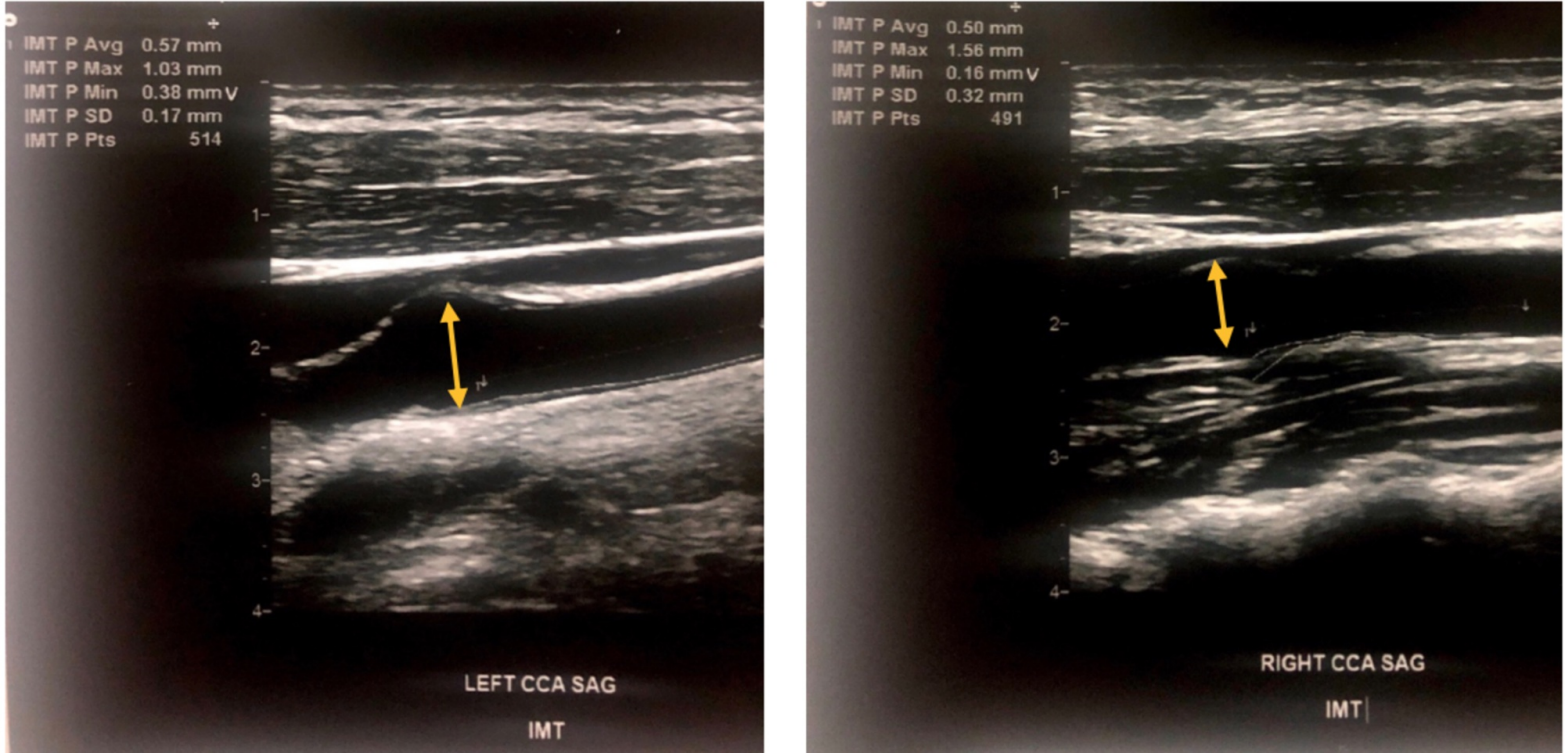
Ultrasound showing the left common carotid intima-media thickness (left panel) and right common carotid intima-media thickness (right panel) in 2019.

**Figure 4:**
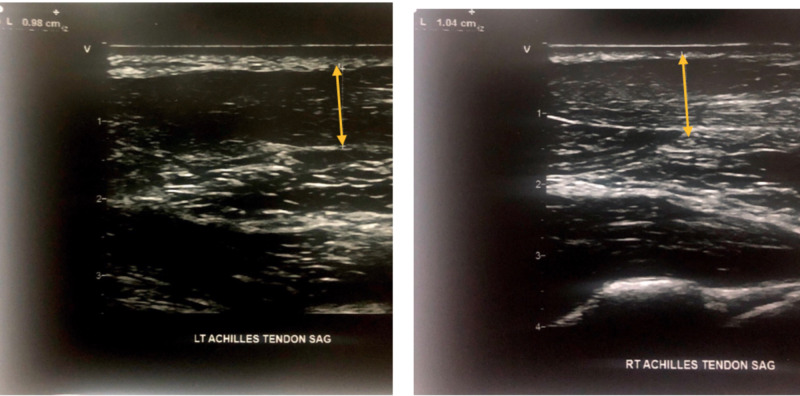
Ultrasound showing the left Achilles tendon thickness (left panel) and right Achilles tendon thickness (right panel) in 2019.

He was maintained on aggressive lipid-lowering regimen and monitored at interval clinic visits. At a two-year follow-up in May 2019, ultrasound of bilateral common carotids showed CIMT to be 0.5mm on right and 0.57mm on left, with a mean CIMT reduction of 38% (Figure 3). Ultrasound of bilateral Achilles tendon showed ATT to be 1.04cm on the right and 0.98cm on the left (no significant change) (Figure 4). Lipid panel at that time also revealed a marked 57% reduction in total cholesterol with a value of 180mg/dl and 73% reduction in LDL-c with a value of 102 mg/dl. Results of CIMT, ATT, and lipid panel are summarized in Table 1.

**Table 1:** Reduction on carotid intima-media thickness, total cholesterol, and low-density lipoprotein-c at a two-year interval.

	2017	2019	% Reduction
Right CIMT	0.91mm	0.5mm	45%
Left CIMT	0.78mm	0.57mm	27%
Right ATT	0.91cm	1.13cm	-
Left ATT	1.11cm	0.94cm	-
Total cholesterol	419mg/dl	180mg/dl	57%
LDL-c	346mg/dl	95mg/dl	73%

## Discussion

FH is an autosomal dominant disorder that leads to premature atherosclerosis and early onset ASCVD. It can remain undiagnosed until a life-threatening cardiovascular event occurs. Without early detection and appropriate treatment, many patients remain undiagnosed and opportunity for primary prevention is missed. Common genetic mutations seen in FH include LDL receptor gene, apolipoprotein B (apoB) gene, and PCSK9 gene, all of which lead to defective metabolism of LDL-c and elevated plasma levels of LDL-c [6]. Based on large genetic studies, the prevalence of heterozygous FH is noted to be one in 220 [6]. Due to the founder effect and higher mutation rates, the prevalence of FH is noted to be higher in certain populations. Several clinical criteria have been developed to diagnose FH, which include LDL-c level, family history, and physical exam findings of xanthelasmas (cholesterol deposits in skin or eyelids) or corneal arcus. LDL-c greater than or equal to 190mg/dL raises suspicion for the disease, although lower levels in patients with strong family history and/or pathognomonic physical exam findings may aid in diagnosis. 

Reducing LDL-c below target level is the primary goal of treatment which can be achieved with maximally tolerated statin therapy, along with the addition of ezetimibe and PCSK9 inhibitor therapy. Aggressive lipid-lowering drugs, such as PCSK9 inhibitors are human monoclonal antibodies that prevent LDL receptor degradation and reduce serum LDL cholesterol by increasing its uptake by the hepatocytes [7]. It has been shown that in heterozygous FH patients, high PCSK9 levels are associated with carotid atherosclerosis as measured by CIMT [8]. In the GLAGOV trial that included 968 patients, the PCSK9 inhibitor evolocumab resulted in ~1% reduction of atheroma plaque volume measured by intravascular ultrasound [9]. In another study presented at the European Society of Cardiology, the addition of evolocumab in 56 patients with dyslipidemia resulted in rapid and significant CIMT plaque reversal at six months interval [10]. Prior meta-analysis has shown for every 0.1mm increase in intimal thickness, the risk of myocardial infarction is increased by 10-15% [11], suggesting our patient who saw a mean intimal thickness reduction of 0.31mm had a marked mean risk reduction of 30-45%. Aggressive lipid-lowering therapy with PCSK9 inhibition has shown regression of CIMT, and inconsistent regression of ATT. We report a case which suggests that PCSK9 inhibition is highly efficient in reducing CIMT plaque burden. Ultrasound-guided measurement of CIMT in conjunction with advanced lipid panel measurements can be used to monitor effects of PCSK9 inhibition on reducing ASCVD.

## Conclusions

Our case highlights a high-risk patient with virulent heterozygous FH who experienced a marked reduction in carotid artery plaque burden, total cholesterol, and LDL-c with PCSK9 inhibition therapy. He did not have any significant changes in his ATT. At a two-year follow-up, we used the ultrasonographic CIMT plaque reduction to predict his future ASCVD risk reduction and to demonstrate the efficacy of PCSK9 inhibition. Ultrasound-guided measurement of CIMT is a highly accessible and noninvasive tool that can be used to stratify ASCVD progression in high-risk patients such as our case, as well as to quantify PCSK9 inhibitor effects on atherosclerotic plaques.
